# The Association between Dietary Habits and Rapid Postoperative Recovery of Rotator Cuff Repair

**DOI:** 10.3390/nu15214587

**Published:** 2023-10-28

**Authors:** Jiaxin Liu, Wei Wang, Zhifeng Wang, Qingyun Wu, Yunli Zhu, Weicheng Wu, Qi Zhou

**Affiliations:** 1 Department of Orthopedics, Changzheng Hospital, Naval Medical University, Shanghai 200003, China; 22112030029@m.fudan.edu.cn (J.L.); czgjww2016@163.com (W.W.); joint-zhu@126.com (Y.Z.); 2 State Key Laboratory of Genetic Engineering and MOE Key Laboratory of Contemporary Anthropology, Human Phenome Institute, School of Life Sciences, Fudan University, Shanghai 200438, China; 23112030035@m.fudan.edu.cn (Z.W.); 23212030016@m.fudan.edu.cn (Q.W.); 3 Rugao Research Institute of Longevity and Aging, Fudan University, Rugao 226500, China

**Keywords:** rotator cuff repair, postoperative recovery, rapid recovery, dietary habits, eosinophilic granulocyte

## Abstract

Some nutritional factors have been suggested to improve postoperative outcomes in rotator cuff (RC) repair, but dietary effects on the recovery speed after the surgery remain undefined. To investigate the potential roles of dietary habits in this context, we analyzed the 12-month follow-up data of 55 patients with RC repair and found that these patients could be categorized into a rapid recovery group (n = 35) and slow recovery group (n = 20) according to their postoperative recovery patterns. Group-based logistic analysis revealed that habitual intakes of meat (OR = 1.84, 95%CI, 1.22–2.76, *p* = 0.003), fruits (OR = 2.33, 95%CI, 1.26–5.67, *p* = 0.01), and wheat-flour foods (OR = 1.62, 95%CI, 1.2–2.25, *p* = 0.002) were significantly associated with rapid recovery. Moreover, among all intakes of wheat-flour foods, intakes of steamed and boiled flour products were also associated with rapid recovery. Further mediation analysis showed that eosinophilic granulocytes (EOs) significantly mediated the association between rapid RC recovery and the habitual intakes of meat (mediation proportion = 17.5%, P-mediation < 0.0001), fruits (17.9%, *p* < 0.0001), and wheat-flour foods (11.4%, *p* < 0.0001). Thus, our study suggests that certain dietary habits play beneficial roles in the context of postoperative recovery for RC repair.

## 1. Introduction

Rotator cuff (RC) disease is a musculoskeletal system disorder with a high prevalence and second only to chronic knee pain in our society’s burden of musculoskeletal disease. During the initial phases of an injury, a considerable proportion of these tears do not exhibit any symptoms; nevertheless, they have the potential to develop into a degenerative condition and subsequently evolve into a chronic tear. If left untreated, it that can progress in severity and tear size, causing pain, dysfunction, and even disability for afflicted people. The American Academy of Orthopedic Surgeons (AAOS) guidelines recommend surgical repair over conservative treatment for patients with symptomatic, chronic, RC, full-thickness tears, which is commonly performed worldwide [1,2]. Each year, at least 30 million people worldwide are affected by RC injury, with 4.5 million patients diagnosed with RC injury in the United States [3]. Almost 250,000 of these cases are treated via surgical repair, generating over USD 3 billion in costs annually [4]. Despite the recent advantages obtained using patch materials and therapeutic approaches in improving clinical outcomes for arthroscopically repaired RCs, complete recovery following arthroscopic repair of large and very large RC tears is still unattainable [5,6].

Several factors have been reported to be associated with better outcomes after operative treatment of RC, including age [7], stiffness [8], gender [9], fatty infiltration [10], tear size [11], and comorbidities [12]. Although various nutrients, such as proteins, amino acids (leucine, arginine, glutamine), vitamins C and D, manganese, copper, zinc, and phytochemicals, have also been suggested to contribute to improving RC tendon growth and healing [13], less preliminary evidence supports the idea that they influence the postoperative recovery of RC repair. Gumina et al. showed the long-term effectiveness of hydrolyzed collagen, methylsulfonyl–methane, and arginine for pain reduction in patients after RC repair [14]. Curcumin, Boswellia serrata, and bromelain were also found to be associated with pain improvement [14,15,16,17]. Some studies have also revealed that leucine and essential fatty acids are beneficial in the improvement of disability and pain outcomes in patients with RC disease [15,18,19]. However, most of these studies have focused on the effects of dietary supplements, while a limited number of studies have investigated the potential impact of dietary habits on RC tendon health, and even fewer have investigated surgical outcomes.

Therefore, in this study, we aimed to determine whether dietary habits influence postoperative pain and functional outcomes relating to RC repair, identify potential beneficial dietary factors, and investigate potential mediators regulating the effects of these factors on recovery. Our results can guide the decision to make early dietary adjustments in high-risk populations and may serve to instruct clinicians and patients in optimizing postoperative diets for the improvement of nutritional intake and recovery outcomes following RC repair.

## 2. Materials and Methods

### 2.1. Participants

Between February 2019 and November 2021, the Shanghai Rotator Cuff Repair (SRCR) cohort study recruited patients 40 years of age and older with symptomatic RC tears (for at least 4 weeks) undergoing operative treatment at the Department of Orthopedics, Changzheng Hospital, Naval Medical University, Shanghai, China, and to participate in follow-up survey. The exclusion criteria included a current shoulder fracture, prior shoulder surgery (on the index shoulder), and active cervical radiculopathy (elicited as neck pain radiating to the shoulder/arm/hand). Although this analysis was performed in patients undergoing RC repair, the entire SRCR cohort recruited patients with and without tears, along with those undergoing operative and non-operative treatments. A total of 55 patients were ultimately included in this study.

### 2.2. Demographic Questionnaire and Immunity Marker Measurement

Participants completed a baseline questionnaire before surgery, and three outcome questionnaires following third, sixth, and twelfth after RC repair surgeries. The questionnaire elicited comprehensive and structured information on patients’ demographics, symptoms, smoking/alcohol habits, dietary habits, pain, and shoulder functions. Blood samples were collected and sent to the laboratory for testing immediately after sampling by professional physicians. In addition, the patient should be seated in the correct position for blood collection with the elbow on a firm operating table and adequate exposure of the elbow vein. Also, the patient was asked to clench his fist, the patient’s arm was examined, and a vein that was most suitable for blood collection was selected. A pressure belt was attached 5 to 10 cm above the suitable blood collection site to fully expose the veins to be sterilized for blood collection by puncture. The blood detection items included blood routine indexes, the erythrocyte sedimentation rate (ESR), coagulation indexes, and blood biochemical indexes. All indexes were categorized into “normal” and “abnormal” types according to normal references in analysis.

### 2.3. Food Frequency Questionnaire

A Food Frequency Questionnaire (FFQ) is a scale used for the qualitative assessment of dietary intake patterns over a long and imprecise time period. It is widely employed in academic research to explore the association between dietary factors and chronic diseases. This instrument is particularly favored due to its cost-effectiveness and capacity to accurately assess habitual dietary patterns [20,21]. The simplified FFQ scale is considered more suitable for assessing food consumption and nutritional intake in large-scale surveys in China. It contains 17 questions about the consumption of food, including meat (pork, beef, mutton, and poultry), fish, legumes and legume products, fresh vegetables, milk and dairy products, fruits, potatoes, salted vegetables, smoked food, rice, wheat flour, other grains, vegetable oils, animal fats, and liquor (wine and beer). Consumption frequency is defined as (1) never, (2) less than once per month, (3) 1–3 times per month, (4) once per week, (5) 2–3 times per week, (6) 4–5 times per week, (7) once per day, (8) 2 times per day, and (9) 3 times or more per day [22]. All patients had an almost daily intake of vegetable oil, and none drank wine and ate other grains, so these three items were excluded in the analysis.

### 2.4. Evaluation of Pain and Shoulder Function

The Visual Analog Scale (VAS) is a unidimensional tool utilized to assess pain intensity, and its application has been extensive across various adult populations. The score is ascertained by employing a ruler to measure the distance (in millimeters) on the 10 cm line between the reference point denoting “no pain” and the mark made by the patient, thereby yielding a spectrum of scores ranging from 0 to 100. The clinical assessments were performed both pre- and postoperation under the guidance of professional doctors. Pain was assessed using the VAS questionnaire, and it includes the states of rest and activity. It is based on the distribution of pain VAS scores in postsurgical patients who described their postoperative pain intensity as none, mild, moderate, or severe. The higher the number, the higher the severity of the pain [23]. The UCLA Shoulder Score was initially introduced in 1981 within the publication of Clinical Orthopaedics and Related Research, with the primary objective of evaluating clinical outcomes subsequent to total shoulder arthroplasty. Here, shoulder function and patient satisfaction were evaluated using the University of California, Los Angeles (UCLA) shoulder scale. Patients provided subjective evaluations of pain and functional activity, while doctors conducted objective evaluations of shoulder range of motion and muscle strength. The UCLA scale is a 35-point scale with 10 points for pain, 10 points for function, and 5 points each for motion, strength, and patient satisfaction. When evaluating the degree of shoulder pain, shoulder function, and range of motion of the shoulder joint, it is found that it may not be exactly settled to a specific score. As a flexible treatment, the middle value between the two values can be given. In terms of the degree of shoulder pain, and shoulder joint function, each aspect can be divided into four grades: excellent, good, fair, and poor. A score of 8 or more is considered excellent, a score greater than 6 is considered good, a scores greater than 4 was considered fair, and a score of less than 3 was considered poor. Excellent (34 to 35 points), good (28 to 33 points), fair (21 to 27 points) and poor (20 or less points) in the total score. Excellent and good scores (>28 points) were considered satisfactory; fair and poor scores (≤28 points) were considered unsatisfactory [24]. We divided the patients into a rapid recovery group and slow recovery group according to their recovery rates. The recovery rate is defined as follows:

$$\mathrm{recovery}\;\mathrm{ratio}=\frac{{\mathrm{Score}}^{3\mathrm{rd}\;\mathrm{month}}-{\mathrm{Score}}^{0\mathrm{th}\;\mathrm{month}}}{{\mathrm{Score}}^{6\mathrm{th}\;\mathrm{month}}-{\mathrm{Score}}^{3\mathrm{rd}\;\mathrm{month}}}$$

### 2.5. Statistical Analysis

Data are expressed as the mean and SD for categorical variables. For group comparisons, a two-sample *t*-test and chi-square test were used. To determine changes within or between groups for all variables, logistic regression analysis for repeated measurements was performed to account for the correlation from the same subject, with adjustment for the baseline value. The mediation package and linear regression equation were used to fit and obtain the values of its direct, indirect, and total effects. In the analysis, habitual diet was the independent variable, and the dependent variable was the rapid postoperative recovery for the rotator cuff. The standardized coefficients, standard errors, and 95% confidence intervals are reported. All statistical analyses were performed using R (version 4.2.2; R Foundation for Statistical Computing), all *p*-values and 95% CIs are two-sided, and *p* < 0.05 was considered to indicate a statistically significant difference or association.

## 3. Results

### 3.1. Research Design and Demographic Characteristics

**Figure 1:**
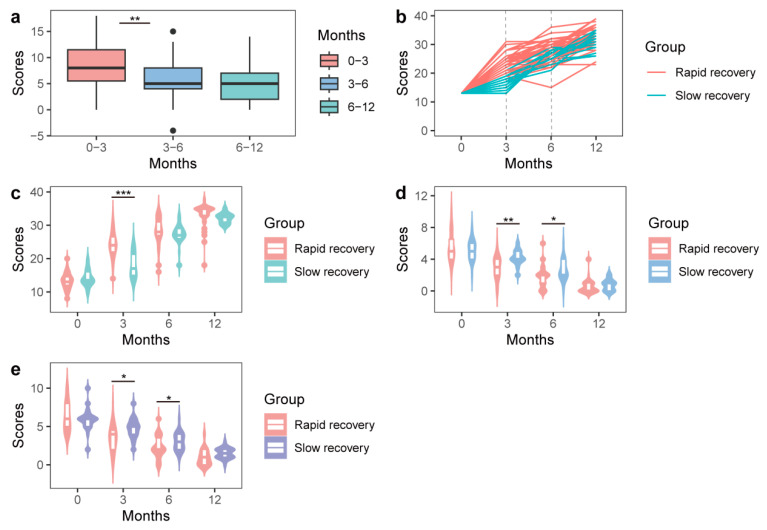
Shoulder functions at different time points. (**a**) UCLA shoulder scores for all patients at different time points. (**b**) Patient grouping according to the changing patterns of the UCLA shoulder scores postoperatively. (**c**–**e**) Differences in the UCLA shoulder scores (**c**) and VAS scores ((**d**) activity and (**e**) rest) between the rapid recovery and slow recovery groups at different time points. * Student’s *t*-test, *p* < 0.05, ** *p* < 0.001, *** *p* < 0.0001.

A total of 55 patients were included in this study (23 males/32 females). The average age of the patients was 53.16 (SD = 12.52) years, and the average BMI was 24.19 (SD = 2.91). The patients’ shoulder pain and functions were evaluated before the surgery and at three, six, and twelve months postoperation. The VAS scores gradually decreased and UCLA shoulder scores increased with time following RC repair that reflecting shoulder pain and functions. On the other hand, the changes in UCLA shoulder scores conferred significant differences during the recovery period 6 months postoperatively, rather than after 6 months (Figure 1a). Based on the changing pattern of UCLA shoulder scores between the third and sixth months postoperation, we divided participants into a “rapid recovery group” (n = 35) with a recovery ratio > 1, and a “slow recovery group” (n = 20) with a recovery ratio ≤ 1 (Figure 1b). After grouping, significant differences in either the VAS or UCLA shoulder scores remained between the rapid recovery and slow recovery groups at the third month postoperatively (Figure 1c–e). The characteristics of the baseline and follow-up data, including age, gender, BMI, occupation, marital status, smoking, drinking, VAS score, and UCLA shoulder score, are shown in Table 1. The average age of the patients was 53.16 (SD = 12.52) years, and all were laborers. The patients included male (n = 23) and female (n = 32) patients. Their average BMI was 24.19 (SD = 2.91), 21.82 percent were smokers, and 14.55 percent were drinkers. The 89.09 percent of patients were married. The UCLA shoulder scores increased and VAS scores decreased with postoperative recovery.

### 3.2. Rapid Postoperative RC Recovery Was Significantly Associated with Habitual Intakes of meat, Fruits, and Wheat-Flour Foods

**Figure 2:**
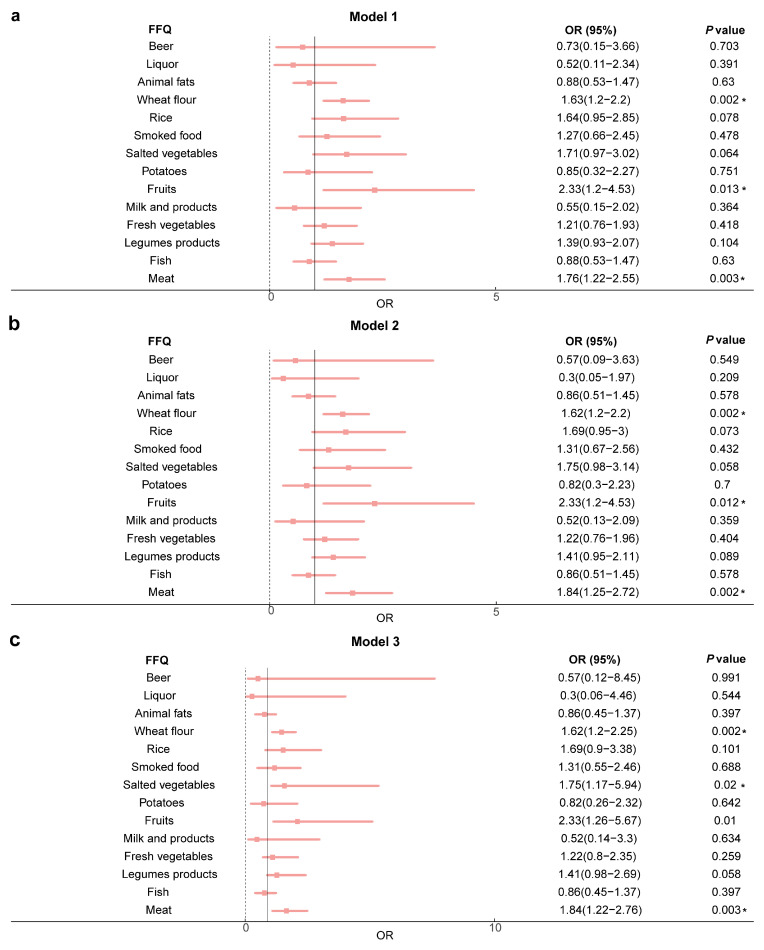
The association between rapid postoperative RC recovery and habitual intakes of meat, fruits, and wheat-flour foods. (**a**) Model 1—no adjustment. (**b**) Model 2—adjusted for age and gender. (**c**) Model 3—adjusted for age, gender, BMI, smoke, and marriage. Abbreviations: FFQ, Food Frequency Questionnaires; OR, odds ratio; BMI, body mass index. * Logistic regression analysis, *p* < 0.05.

We performed logistic analysis on the association between rapid RC recovery and scores for 14 questions about food consumption from the simplified FFQ scale. Using the crude model showed that rapid postoperative RC recovery was significantly associated with habitual intake of meat (OR = 1.76, 95%CI, 1.22–2.55, *p* = 0.003), fruits (OR = 2.33, 95%CI, 1.2–4.53, *p* = 0.013), and wheat-flour foods (OR = 1.63, 95%CI, 1.2–2.2, *p* = 0.002) (Figure 2a). After adjustment for age and gender, these associations were still significant, such as meat (OR = 1.84, 95%CI, 1.25–2.72, *p* = 0.002), fruits (OR = 2.33, 95%CI, 1.2–4.53, *p* = 0.012) and wheat-flour foods (OR = 1.62, 95%CI, 1.2–2.2, *p* = 0.002) (Figure 2b), and remained significant in the model (*p* = 0.002, *p* = 0.012 and *p* = 0.002) fully adjusted for age, gender, BMI, marital status, and smoking status (Figure 2c). These results indicate that habitual intakes of meat, fruits, and wheat-flour foods are significantly associated with rapid postoperative RC recovery.

### 3.3. Rapid Postoperative RC Recovery Was Closely Related to Chinese Habitual Intakes of Steamed and Boiled Products

**Figure 3:**
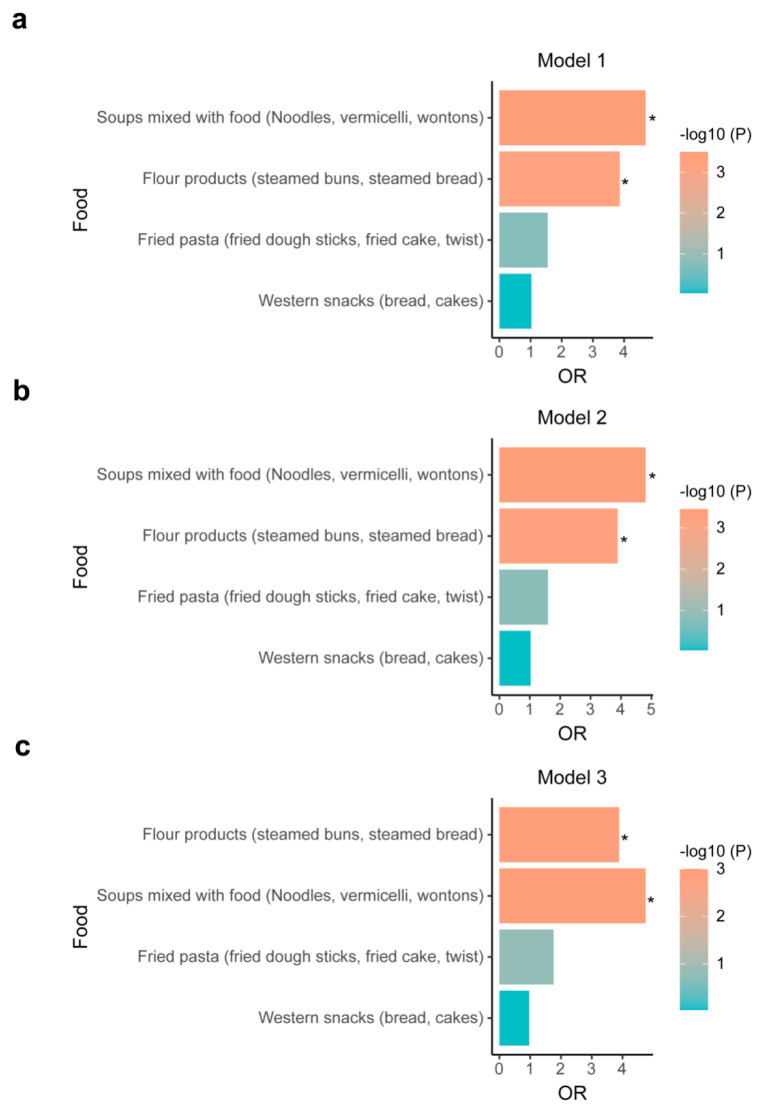
The association between rapid postoperative RC recovery and habitual intakes of four types of wheat-flour foods. (**a**) Model 1—no adjustment. (**b**) Model 2—adjusted for age and gender (**c**) Model 3—adjusted for age, gender, BMI, smoke, and marriage. Abbreviations: FFQ, Food Frequency Questionnaires; OR, odds ratio; BMI, body mass index. * Logistic regression analysis, *p* < 0.05.

Studies on the types of potentially beneficial wheat-flour foods for postoperative recovery are limited. Therefore, we analyzed the association between rapid recovery in RC repair and four types of wheat flour foods commonly consumed in China, including “steamed flour products such as steamed buns and steamed bread”, “noodles, vermicelli and wontons of soup-mixed foods”, “bread and cakes of western snacks”, and “fried dough sticks, cake and twist of fried pasta”. We concluded that without any adjustment for confounding factors, rapid recovery was significantly associated with “steamed flour products such as steamed buns and steamed bread” (OR = 3.86, 95%CI, 1.79–8.35, *p* = 0.0006) and “soup-mixed foods like noodles, vermicelli and wontons” (OR = 4.69, 95%CI, 2.02–10.87, *p* = 0.0003) (Figure 3a). In the model adjusted for gender and age (*p* = 0.001 and *p* < 0.001) (Figure 3b) and the fully adjusted model (*p* = 0.001 and *p* = 0.001) ((Figure 3c), these associations remained significant, suggesting that these Chinese-style flour products had beneficial effects on RC recovery.

### 3.4. EOs Mediated the Association between Rapid Postoperative RC Recovery and Dietary Habits

**Figure 4:**
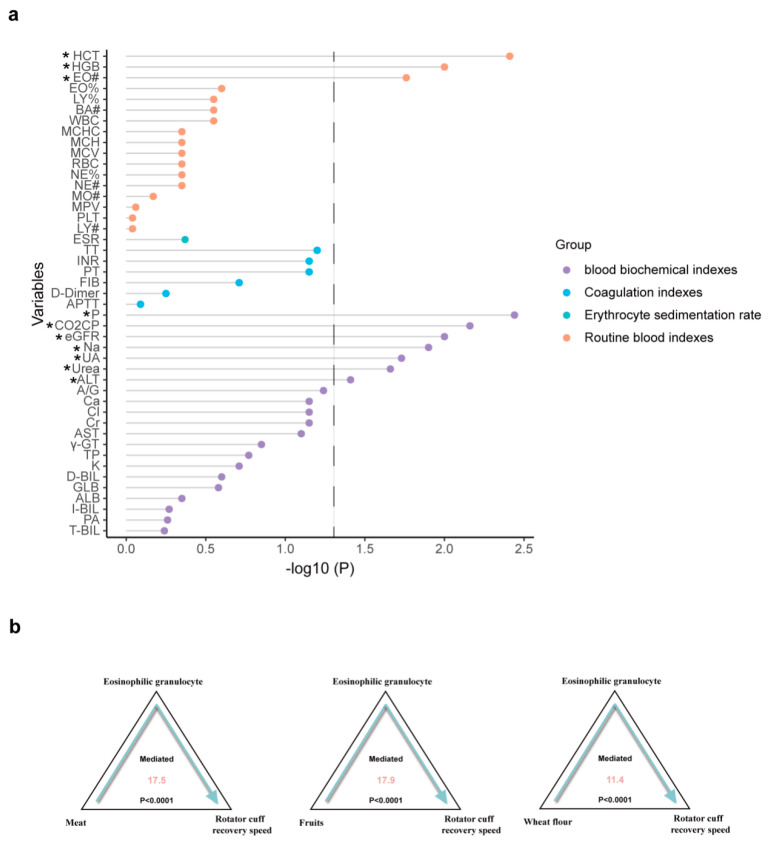
The mediation analysis between rapid recovery and dietary habits. (**a**) The association between rapid postoperative RC recovery and blood indicators. (**b**) The mediation analysis between rapid recovery and habitual intakes of meat, fruits, and wheat-flour foods. * p < 0.05. Abbreviations are listed in Supplementary Table S1.

To identify the possible mediating factors underlying the association between RC recovery speed and the beneficial dietary habits observed above, we utilized blood indicators, sunch as blood routine indexes, the erythrocyte sedimentation rate, coagulation indicators, and blood biochemical criteria as the mediating factors for mediation analysis. For the hypothesized mediation model, the independent variable was habitual diet and the dependent variable was the postoperative recovery speed for the rotator cuff. First, we performed correlation analysis and determined that aberrant blood levels of EO, HCB, HCT, ALT, urea, UA, Na, P, CO2CP, and eGFR were significantly associated with rapid RC recovery (Figure 4a). Further mediation analysis on these mediating effect of the rapid recovery and habitual intakes of meat (mediation proportion = 17.5%, P-mediation < 0.0001), fruits (mediation proportion = 17.9%, P-mediation < 0.0001) and wheat flour foods (mediation proportion = 11.4%, P-mediation < 0.0001) (Figure 4b). It reveals that EO was a potential mediator of the association.

## 4. Discussion

With the increase in research in recent years, various adjuvant strategies and postoperative measures improving the surgical outcomes in RC injury have been suggested [7,8,9,10,11,12]. Among these measures, the administration of some nutritional or dietary supplements was reported to benefit postoperative recovery [13,25,26]. For example, increasing dietary protein may be beneficial after an injury, in terms of both attenuating muscle atrophy and promoting repair [27]. Furthermore, increasing dietary glycine intake can promote collagen synthesis, thus producing beneficial effects on tendon healing [28]. Additionally, the intake of a diet rich in glucosamine and chondroitin sulfate was associated with lower tissue inflammation and increased collagen synthesis during tenotomy [29]. However, there is still no clear dietary recommendation for patients with RC repair due to the lack of studies on the association between dietary habits and recovery speed [15]. Therefore, we collected and analyzed the follow-up data of 55 patients with RC repair and found that this small population can be divided into two groups according to the recovery speed of shoulder function, especially between the third and sixth months postoperation. The phenomenon of rapid RC repair recovery in 6 months is consistent with previous reports showing that patients who have undergone rotator cuff repair experience around 75% functional recovery at 6 months after surgery [30]. Based on the grouping results, our data highlight significant associations between rapid postoperative recovery for RC repair and several dietary habits, including the habitual intake of meat, fruits, and wheat flour. Further analysis on specific types of wheat-flour foods revealed that Chinese-style steaming and boiling flour products were also associated with rapid recovery.

Despite the lack of direct evidence supporting the idea that dietary factors affect rotator cuff recovery in previous reports, numerous studies have proven that dietary habits and supplement intake could regulate tendon health [13,25,26]. Since tendon impairment is one of the major causes of RC injury and tendon health is required for the process of RC recovery [31,32], dietary elements and habits associated with tendon health may also be critical for RC healing. For example, alcohol impacts RC recovery by inhibiting fibroblast proliferation and collagen synthesis, and also causes direct toxic effects and damage to the microvascular system in tendons [33]. Thus, excessive alcohol consumption is an important risk factor for both tendon health and RC tear recovery. Regarding beneficial elements, nutrients, such as protein, amino acids, and vitamins C and D, were reported to improve tendon growth and healing [13]. The combined use of collagen and vitamin C was more effective than a single nutrient [13], suggesting that multiple nutrients, such as those found in meat and fruits, may improve tendon healing, thus promoting RC recovery. On the other hand, it was reported that too little fruit and vegetable intake may be a factor contributing to the poor outcomes for other surgeries, such as renal transplants [34,35]. Our data show that habitual intakes of meat and fruits are associated with rapid RC recovery, strengthening evidence for the beneficial roles of these dietary foods in promoting RC recovery.

In addition to the association for meat and fruits, our data also reveal a strong association between wheat-flour foods and rapid RC recovery, outlining their potential benefits. Intriguingly, we found that steamed and boiled wheat-flour foods, instead of fried flour foods or Western desserts, were associated with rapid RC recovery postoperatively. Since Chinese-style steamed or boiled flour products, such as steamed buns with meat stuffing, wontons, dumplings, and noodle soups, are generally made with both flour and meat and considering the significant association between meat and rapid RC recovery observed above, these results suggest that the combined intake of flour and meat may accelerate postoperative RC recovery.

Alongside dietary habits, other factors, such as inflammation, metabolism, and hepatic and renal function, confer impacts on postoperative recovery [36,37,38]. Since dietary habits were reported to influence factors such as inflammation [39], metabolism [40], liver health [41] and kidney [42] function, we speculated that the potential effects of dietary habits on rapid RC recovery may be mediated by any one of these factors. We first used the blood routine parameters and serum biochemical indicators that reflect the statuses of inflammation, metabolism, hepatic function, and renal functions in patients, and explored their correlations with RC recovery patterns. Several routine blood indicators, such as EO, HGB, and HCT, and some blood biochemical indicators, such as ALT, urea, UA, Na, P, CO2CP, and eGFR, were screened, indicating that the statuses of inflammation, metabolism, and hepatic and renal functions are closely related to postoperative RC recovery.

Further mediation analysis indicated that the number of EOs could simultaneously mediate the association of rapid RC recovery with habitual intakes of meat, fruits, and wheat-flour foods. In fact, a large number of studies have shown that EOs are involved in postoperative recovery, and EOs were associated with the outcomes of various types of surgeries [43,44,45]. For example, increased blood EO concentrations can predict surgical intervention for chronic rhinosinusitis, and in both invasive and non-invasive sinus surgery, the number of EOs in patients during or after surgery can be an indicator of recovery [44,45]. EOs can also be used as inflammatory markers to predict the responses of patients undergoing cholangitis surgery [43]. However, EOs in the blood may also be elevated due to the intake of dietary proteins [46,47]. For example, there are eosinophilic gastrointestinal disorders that are usually caused by food protein, and elemental diet therapy using an amino acid-based formula can improve eosinophilic gastroenteritis, potentially reducing the number of EOs [48]. Therefore, these nutrients from meat, fruits, and wheat flour may affect recovery via EOs. As a whole, this evidence supports our speculation that dietary habitual intakes of meat, fruits, and wheat flour may accelerate postoperative RC recovery by regulating the amount of EO. However, the further study will be necessary in the future.

## 5. Limitations

First, the sample size of this study was small and was unable to generate clinical recommendations for patients after RC repair; further studies with larger sample sizes are needed to reach a more robust conclusion. Second, although the simplified FFQ scale used in this study was sufficient to reflect the major dietary pattern, a more detailed scale will make it possible to refine the beneficial nutritional panel, providing better dietary guidance for patients and clinicians. Third, randomized, controlled studies or interventional experiments are required to demonstrate the effects of dietary habits and relevant nutrients. Finally, this study only applied blood indicators for mediation analysis, while dietary habits may also be closely related to secondary metabolites and the microbes in the digestive system, necessitating metabolomics and metagenomics analysis to elucidate the mechanism.

## 6. Conclusions

In conclusion, our study suggests that certain dietary habits may be beneficial for postoperative recovery in RC repair, and EOs might be involved in the process. Our findings underscore the significant impact of dietary habits on RC repair. This approach offers advantages such as lower cost, increased convenience, receptive and minimal side effects. It not only provides valuable guidance for clinicians that will help them to improve patient prognosis, but also has the potential to alleviate the financial burden on patients, expedite their recovery, and enhance their postoperative quality of life.

## Figures and Tables

**Table 1:** Patient characteristics at baseline and follow-up.

Item			Baseline	Follow-Up	
				Rapid Recovery Group (n = 35)	Slow Recovery Group (n = 20)
Age, mean (SD)			53.16 (12.52)	52.46 (11.95)	54.40 (13.67)
Gender, no. (%)	Male		23 (41.82)	15 (42.85)	8 (40)
	Female		32 (58.18)	20 (57.14)	12 (60)
BMI, mean (SD)			24.19 (2.91)	23.64 (2.69)	25.17 (3.10)
Marital status, no. (%)	Yes		49 (89.09)	31 (88.57)	18 (90)
	No		6 (10.91)	4 (11.43)	2 (10)
Drinker, no. (%)	Yes		8 (14.55)	4 (11.43)	4 (20)
	No		47 (85.45)	31 (88.57)	16 (80)
Smoker, no. (%)	Yes		12 (21.82)	5 (14.29)	7 (35)
	No		43 (78.18)	30 (85.71)	13 (65)
Occupation, no. (%)	Professional technologist		0 (0)	0 (0)	0 (0)
	Laborer		55 (100)	35 (100)	20 (100)
UCLA shoulder scores, mean (SD)	Pre-operation		13.64 (2.59)	13.31 (2.56)	14.20 (2.61)
	Third month		22.04 (4.88)	24.23 (4.02)	18.20 (3.81)
	Sixth month		27.75 (4.16)	28.03 (4.64)	27.25 (3.19)
	Twelfth month		32.67 (3.16)	32.97 (3.78)	32.15 (1.53)
VAS scores, mean (SD)	Rest	Pre-operation	5.42 (1.74)	5.57 (1.93)	5.15 (1.35)
		Third month	3.40 (1.45)	2.91 (1.46)	4.25 (0.97)
		Sixth month	2.05 (1.42)	1.71 (1.36)	2.65 (1.35)
		Twelfth month	0.60 (0.78)	0.51 (0.82)	0.75 (0.72)
	Activity	Pre-operation	6.18 (1.83)	6.34 (1.97)	5.90 (1.55)
		Third month	3.93 (1.74)	3.46 (1.79)	4.75 (1.33)
		Sixth month	2.55 (1.40)	2.26 (1.44)	3.05 (1.19)
		Twelfth month	1.16 (1.00)	0.97 (1.10)	1.50 (0.67)

Abbreviations: BMI, body mass index; UCLA, University of California, Los Angeles; VAS, Visual Analogue Scale.

## Data Availability

All data are fully available without restriction.

## Supplementary Materials

The following supporting information can be downloaded at: https://www.mdpi.com/article/10.3390/nu15214587/s1, Table S1: Meanings of the abbreviations for hematological indicators.
