# Supplementary material for: The Association between Dietary Habits and Rapid Postoperative Recovery of Rotator Cuff Repair
Source: Nutrients. 2023 Oct 28;15(21):4587. doi: 10.3390/nu15214587 (PMC10648498; doi:10.3390/nu15214587)
Supplement: Supplementary file 1 [file nutrients-15-04587-s001.zip › nutrients-2639365-supplementary.pdf]

Table S1. Meanings of the abbreviations for hematological indicators.

Abbreviation	Meaning
**Routine blood indexes**	
HCT	hematocrit
HGB	hemoglobin
EO#	the number of eosinophilic granulocyte
EO%	the percent of eosinophilic granulocyte
LY%	the number of lymphocyte
BA#	the number of basophilia
WBC	white blood cell
MCHC	mean corpusular hemoglobin concerntration
MCH	mean corpusular hemoglobin
MCV	mean corpusular volume
RBC	red blood cell
NE%	the percent of neutrophilic granulocyte
NE#	the number of neutrophilic granulocyte
MO#	the number of macrophage
MPV	mean platelet volume
PLT	platelet count
LY#	the number of lymphocyte
**Erythrocyte sedimentation rate**	
ESR	erythrocyte sedimentation rate
**Coagulation indexes**	
TT	thromboplastin time
INR	international normalized ratio
PT	prothrombin time
FIB	fibrinogen
APTT	activated partial thromboplastin time
**Blood biochemical indexes**	
P	phosphorus
CO2CP	carbondioxide combining power
eGFR	estimated glomerular filtration rate
Na	sodium
UA	uric acid
ALT	alanine amiotransferase
A/G	albumin/globulin
Ca	calcium
Cl	chloride
Cr	creatinine
AST	aspartate aminotransferase
γ-GT	γ-glutamyl transpeptadase
TP	total protein
